# CaMKII inhibitor KN‐93 impaired angiogenesis and aggravated cardiac remodelling and heart failure via inhibiting NOX2/mtROS/p‐VEGFR2 and STAT3 pathways

**DOI:** 10.1111/jcmm.17081

**Published:** 2021-11-29

**Authors:** Yajuan Ni, Jie Deng, Hongyuan Bai, Chang Liu, Xin Liu, Xiaofang Wang

**Affiliations:** ^1^ Department of Cardiology The Second Affiliated Hospital of Xi’an Jiaotong University Xi'an China

**Keywords:** angiogenesis, CaMKII, cardiac remodelling, heart failure, KN‐93

## Abstract

Persistent cardiac Ca^2+^/calmodulin‐dependent Kinase II (CaMKII) activation was considered to promote heart failure (HF) development, some studies believed that CaMKII was a target for therapy of HF. However, CaMKII was an important mediator for the ischaemia‐induced coronary angiogenesis, and new evidence confirmed that angiogenesis inhibited cardiac remodelling and improved heart function, and some conditions which impaired angiogenesis aggravated ventricular remodelling. This study aimed to investigate the roles and the underlying mechanisms of CaMKII inhibitor in cardiac remodelling. First, we induced cardiac remodelling rat model by ISO, pre‐treated by CaMKII inhibitor KN‐93, evaluated heart function by echocardiography measurements, and performed HE staining, Masson staining, Tunel staining, Western blot and RT‐PCR to test cardiac remodelling and myocardial microvessel density; we also observed ultrastructure of cardiac tissue with transmission electron microscope. Second, we cultured HUVECs, pre‐treated by ISO and KN‐93, detected cell proliferation, migration, tubule formation and apoptosis, and carried out Western blot to determine the expression of NOX2, NOX4, VEGF, VEGFR2, p‐VEGFR2 and STAT3; mtROS level was also measured. In vivo, we found KN‐93 severely reduced myocardial microvessel density, caused apoptosis of vascular endothelial cells, enhanced cardiac hypertrophy, myocardial apoptosis, collagen deposition, aggravated the deterioration of myocardial ultrastructure and heart function. In vitro, KN‐93 inhibited HUVECs proliferation, migration and tubule formation, and promoted apoptosis of HUVECs. The expression of NOX2, NOX4, p‐VEGFR2 and STAT3 were down‐regulated by KN‐93; mtROS level was severely reduced by KN‐93. We concluded that KN‐93 impaired angiogenesis and aggravated cardiac remodelling and heart failure via inhibiting NOX2/mtROS/p‐VEGFR2 and STAT3 pathways.

## 1 INTRODUCTION

The prevalence of heart failure (HF) continued to rise over time and was a global difficult problem, and projections showed that the prevalence of HF would increase 46% from 2012 to 2030, resulting in >8 million people ≥18 years of age with HF in the United States.^1^ Although great progress has been made in drug therapy of HF, the effect was limited, and it was necessary to explore new and effective drug targets.

Cardiac remodelling was an important pathological basis of HF and was characterized by myocardial hypertrophy, apoptosis, interstitial fibrosis and reduced heart function.^2^ In recent years, calcium/calmodulin‐dependent protein kinase II (CaMKII) caused wide attention. Studies found that the activation and expression of CaMKII were increased in HF human and animal model,^3^, ^4^ and CaMKII signallings were essential to cardiac remodelling in pressure‐overload mice, or mediated cardiac hypertrophy of rats induced by isoproterenol (ISO). HF was exacerbated in cardiac‐specific CaMKIIdeltaC transgenic mice, inhibition of CaMKII reversed cardiac dysfunction in a genetic model of dilated cardiomyopathy,^5^, ^6^, ^7^, ^8^, ^9^ and some strategies which inhibited CaMKII suppressed cardiac hypertrophy,^10^, ^11^ and a recent study indicated that chronic CaMKII inhibition with KN‐93, a widely used inhibitor of CaMKII, improved cardiac function and cardiac remodelling in HF mice induced by pressure‐overload.^12^ It seemed that CaMKII inhibition was an effective drug to reverse cardiac remodelling and improve heart function.

Of note, CaMKII was an important mediator for the ischaemia‐induced coronary angiogenesis, in which process, CaMKII played a key role in the trigger of vascular endothelial cell growth factor (VEGF) expression, and the inhibitor of CaMKII KN‐93 suppressed the proliferation and migration of cardiac endothelial cells and inhibited angiogenesis.^13^ A newest study demonstrated that cross‐talk between STAT3 and CaMKII mediated endothelial angiogenesis,^14^ and STAT3 was also a crucial factor to promote angiogenesis.^15^, ^16^ Recently, a lot of new evidence confirmed that angiogenesis was contributed to the inhibition of cardiac remodelling and improvement of heart function, beta‐AR blocker metoprolol promoted angiogenesis in hypertrophied myocardium and reduced hypertrophy, and cardiac function of TAC‐induced HF mice was improved by promoting angiogenesis,^17^ ivabradine and a microRNA attenuated cardiac hypertrophy and remodelling via promoting angiogenesis after myocardial infarction,^18^, ^19^ and some conditions which impaired angiogenesis thus promoted or aggravated ventricular remodelling and HF.^20^, ^21^, ^22^ It seemed that inhibition of CaMKII would aggravate cardiac remodelling and HF progression.

So, did inhibitor of CaMKII reverse myocardial remodelling? If not, what were the underlying mechanisms?

The mechanisms of angiogenesis were complex, excepted for STAT3 pathway, mitochondrial reactive oxygen species (mtROS) was a critical activating factor for VEGF signalling, and promoted angiogenesis in endothelial cells, NADPH oxidase 2 (NOX2) sensed Nox4‐derived ROS to promote mtROS production and caused tyrosine phosphorylation of VEGF receptor type 2 (p‐VEGFR2) and induced angiogenesis.^23^

There were four known isozymes of CaMKII (α, β, γ and δ)^24^; in myocardial tissue, CaMKIIδ had been linked to arrhythmia,^25^, ^26^ as well as remodelling due to atrial fibrillation and myocardial infarction.^27^ A protective role for CaMKIIδ had been implicated in ischaemia/reperfusion.^28^ Given these latter associations, CaMKIIδ remains a target of high interest in cardiovascular disease,^29^ KN‐93 had inhibitory effects on CaMKIIδ, and it was widely used as an inhibitor of CaMKII, so, we select KN‐93 as tool in our study.

In this study, by using cardiac hypertrophy rat model induced by ISO, we found that KN‐93 reduced the myocardial microvessel density and aggravated cardiac remodelling and HF. In order to reveal the underlying mechanisms, we cultured the HUVECs and studied the effects of KN‐93 on angiogenesis, and we found that KN‐93 suppressed cell proliferation, migration and tubule formation via inhibiting NOX2/mtROS/ p‐VEGFR2 and STAT3 pathways, and also promoted apoptosis of HUVECs.

## 2 MATERIALS AND METHODS

### 2.1 Animals and treatment

Young male Sprague‐Dawley rats (120–140 g) were obtained from Xi'an Jiao tong University Laboratorial Animal Center (Shaanxi, China). The investigation conformed to The Guide for the Care and Use of Laboratory Animals, published by the US National Institutes of Health (NIH publication no.85–23, revised in 1996); The experimental protocols were approved by the local authorities (Biomedical Ethics Committee of Medical Department of Xi'an Jiao tong University). Rat model of HF was established as described previously by us.^30^ ISO (7 mg/kg) was administered once daily by intraperitoneal injection for 2 weeks (defined as ISO group, *n* = 12), the dosage was according to our previously study,^31^ but was reduced. KN‐93 (10 ug/g) was administered once daily in the same manner at 30 min before ISO was treated (defined as KN‐93 group, *n* = 14), lasted for 2 weeks. Control animals were administrated with 0.9% NaCl (Ctrl group, *n *= 12).

### 2.2 Echocardiographic measurements

Echocardiography measurements were performed to evaluate the heart function and structure as described previously by us.^32^ The left ventricular ejection fraction (LVEF), left ventricular fractional shortening (LVFS), left ventricular end‐diastolic dimensions (LVIDd), left ventricular end‐systolic dimensions (LVIDs), interventricular septal thickness in diastole (IVSTd), left ventricular posterior wall thickness in diastole (LVPWTd) and heart rate (HR) were all measured.

### 2.3 Heart weight to body weight ratio (HW/BW)

Rats were weighed before anaesthetization, fresh hearts were isolated and washed, vessels and other redundant tissues were removed, and then, the heart was weighed; HW/BW (mg/100 g) was calculated.

### 2.4 Histological staining

Hearts were isolated from rats under anaesthesia with chloral hydrate, left ventricular tissues were dissected and fixed in 4% paraformaldehyde, embedded in paraffin, and then, the samples were sectioned into 5 μm‐thick slices. Haematoxylin‐eosin (HE) staining and Masson's trichrome were performed. The cardiac collagen volume fraction (CVF), perivascular fibrosis ratio and the microvascular density (MVD) were measured by using Image‐pro Plus 6.0 (Media Cybernetics, Bethesda). Six fields were randomly selected under 200× microscope, counted the number of microvessels and averaged, and then converted into counts/100 μm^2^.

### 2.5 TUNEL staining

Left ventricular tissues were collected, and the TUNEL assay was performed as described previously by us.^30^

### 2.6 Transmission electron microscope

Preparation of specimens was described previously by us^30^ and was observed with transmission electron microscope (H‐7650, Hitachi Limited). The percentage of vacuolization in mitochondria and density of mitochondria were analysed statistically with Image‐Pro Plus 6.0 (Media Cybernetics).

### 2.7 Quantitative PCR

Quantitative PCR was performed as described previously by us.^33^ Specific primer sequences of collagen I used for real‐time PCR were as follows: Collagen1, forward: 5′‐AAACCCGAGGTATGCTTGATCTGTA‐3′, reverse: 5′‐GTCCCTCGACTCCTACATCTTCTGA‐3′; Collagen Ⅲ, forward: 5′‐ACGTAAGCACTGGTGGACAG‐3′, reverse: 5′‐CAGGAGGGCCATAGCTGAAC‐3′; β‐actin, forward: 5′‐ACCCTGAAGTACCCCATGCAG‐3′, reverse: 5′‐ACATGATCTGGGTCATCTTGTGC‐3′. The relative level of mRNA was calculated by normalizing to β‐actin, according to the 2^−ΔΔ^CT method.

### 2.8 Western blot analysis of ANP, β‐MHC, bax, bcl‐2, NOX2, NOX4, VEGF, VEGFR2, p‐VEGFR2 and STAT3 protein expression

Briefly, total protein of left ventricular tissue was extracted using RIPA Buffer (BioRad), which contained Protease Inhibitor Cocktail (Sigma‐Aldrich). BCA method was used to detect the concentration. The sample was fractionated and transferred, then blocked and incubated with ANP, β‐MHC, bax, bcl‐2, NOX2, NOX4, VEGF, VEGFR2, p‐VEGFR2 and STAT3 antibody (diluted 1:200, Santa Cruz, USA), and then incubated with a horseradish peroxidase‐conjugated goat mouse antibody (diluted 1:2,000, Santa Cruz, USA). The relative level of protein was calculated by normalizing mean ray value to β‐actin. A chemiluminescence system (ChemiDoc XRS, BioRad) was used to detect protein bands.

### 2.9 Cell Culture and treatment

HUVEC cells were purchased from BeNa Culture Collection and cultured in ECM medium containing 10% FBS (1.5 mM L‐glutamine, 100 U/ml penicillin and 100 μg/ml Streptomycin) and incubated in 37°C 5% CO_2_ saturated humidity incubator.

### 2.10 Proliferation test

Adherent cells were digested by trypsin, and cell suspension was prepared, counted and diluted the cells to 5 × 10^5^/ml. The cells were inoculated into 96 hole cell culture plate with lower cell density (5 × 10^3^); VEGF at a concentration of 10 ng/ml was added and cultured in 37ºC 5% CO_2_ cell incubator overnight. After cell adhesion, CCK‐8 solution according to the ratio of 5:1 was added, cultured for 2 h in the dark and measured the OD value at 450 nm wavelength for 0 h. Absorbed and discarded CCK‐8 solution in 96‐well plate washed by PBS. In the KN‐93+ISO group, the concentration gradient (1, 10 and 50 uM) of KN‐93 was set, KN‐93 with final concentration of (1 uM, 10 uM and 50 uM) was firstly added separately, and ISO (final concentration of 10 uM) was added after 30 min incubation of ISO, the ISO group was added with ISO10uM, Ctrl group was added with equal volume of medium, and OD values of 24, 48 and 72 h after treatment were measured. Before each measurement, cells of 3 groups were added CCK‐8 solution (5:1) and cultured for 2 h in the dark.

### 2.11 Migration detection

The proliferation of HUVECs was quantified by MTT assay. When the cells were cultured in 6‐well plate for 24 h, a 200 ul tip of pipettor was used to draw evenly straight lines vertically through the holes in the 6‐well plate, 2 lines for each hole, then washed and took pictures at 0 h. Added KN‐93, ISO, the concentration was same as above and cultured for 24 h, took pictures again.

### 2.12 Tubule formation experiments

Five hundred microliter matrix gel (diluted with serum‐free base medium 1:8) was absorbed into a 12‐well plate and put it into the incubator and gel for 30 min; cells were digested to prepare cell suspension, added to 12‐well plate and added KN‐93, ISO; the concentration was same as above. Then put 12‐well plate in an incubator with 37°C and 5% CO_2_, cultured for 48 h and take photographs.

### 2.13 Detection of apoptotic cells by flow cytometry

Early stage of apoptosis, late stage of apoptosis or necrosis, cellular debris and viable cells are identified as annexin V (+)/PI (−), annexin V (+)/PI (+), annexin V (−)/PI (+) and annexin V (−)/PI (−) respectively.

### 2.14 Measurement of mtROS

Mitochondria were isolated and purified of HUVECs using Mitochondria Isolation Kit (Sigma) according to the manufacturer's instructions. In addition, mtROS level was performed as described previously by us.^31^

### 2.15 Statistical analysis

All data were presented as means ± SEM. Comparisons between two groups were performed with Student's *t*‐test and among three groups were compared with one‐way ANOVA followed by Tukey post hoc test for significance. All statistics were determined using SPSS15.0 software (SPSS Inc.). A probability value of *p *< 0.05 is considered significant.

## 3 RESULTS

### 3.1 KN‐93 aggravated cardiac remodelling and HF of ISO rats

**FIGURE 1:**
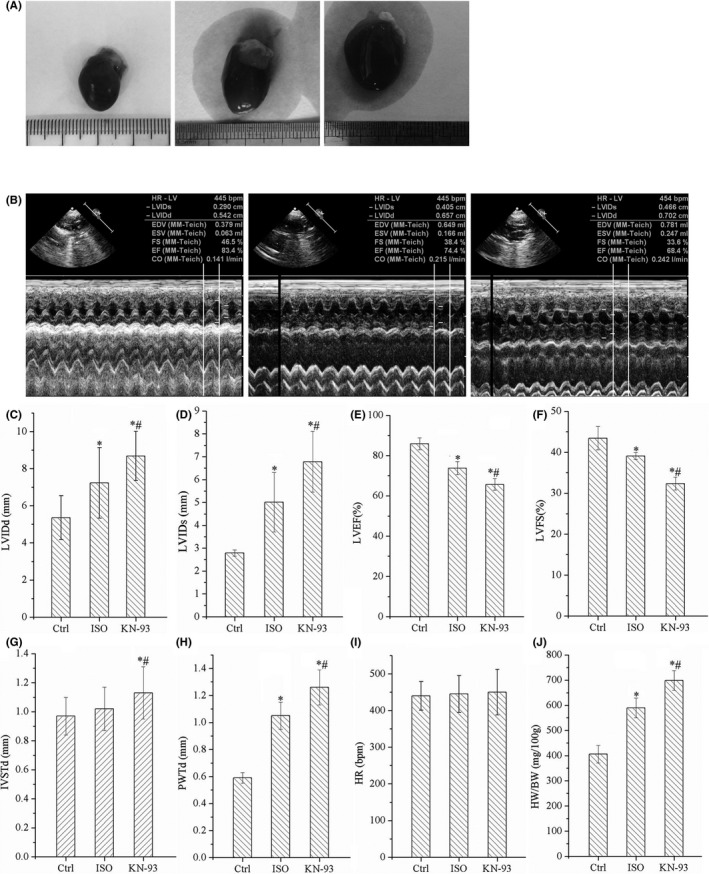
Cardiac remodelling and heart failure were significantly aggravated by KN‐93. (A) KN‐93 aggravated the morphological changes of rats. (B) Representative echocardiographic image of Ctrl, ISO and KN‐93 treated rat. (C–H) KN‐93 obviously reduced LVEF, LVFS, increased LVIDd, LVIDs, IVSTd and LVPWTd. (I) There is no difference among three groups in HR. (J) KN‐93 increased the ratio of HW/BW. All *p < 0.05 versus Ctrl, ^#^ *p *< 0.05 versus ISO. Error bars represent SD

As shown in Figure 1A, B, hearts of ISO rats were obvious hypertrophy and the hypertrophy was aggravated by treatment with KN‐93. Echocardiography data showed that LVEF and LVFS were reduced and LVIDd, LVIDs and LVPWTd were increased in ISO group. However, LVIDd, LVIDs, IVSTd and LVPWTd were increased further in KN‐93 group, and LVEF and LVFS also were reduced further by KN‐93, there was no statistical significance in IVSTd between Ctrl and ISO groups, and there was no significant difference in HR among three groups, and HW/BW was also further increased by KN‐93, as shown in Figure 1C–J. It suggested that KN‐93 aggravated cardiac remodelling and HF of ISO rats.

### 3.2 KN‐93 aggravated cardiac remodelling

**FIGURE 2:**
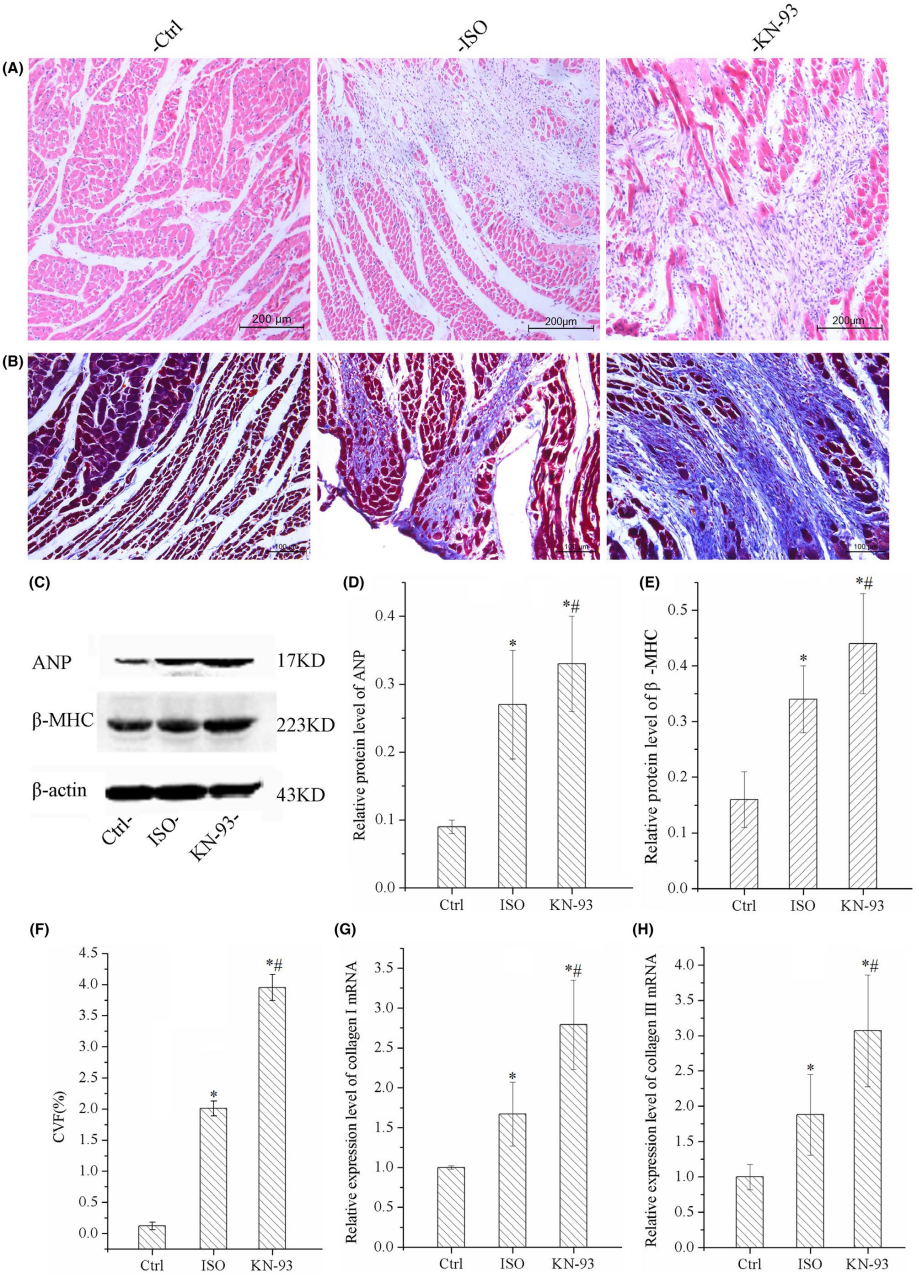
Results of histological staining and WB demonstrated that KN‐93 aggravated left ventricular remodeling of ISO rats. (A) HE staining. (B) Masson's trichrome staining. (C) Representative bands of Western blotting of ANP and β‐MHC. (D and E) KN‐93 up‐regulated protein expression level of ANP and β‐MHC compared with ISO and Ctrl. (F) CVF was increased by KN‐93 compared with ISO and Ctrl. (G and H) KN‐93 up‐regulated the mRNA expression of collagen Ⅰ and collagen Ⅲ. All *p < 0.05 versus Ctrl, ^#^ *p *< 0.05 versus ISO. Error bars represent SD

Results of HE staining indicated that KN‐93 caused more serious inflammatory cell infiltration, myocardial hypertrophy, arranged disorder, degeneration necrosis, and interstitial hyperemia and oedema compared with ISO group and Ctrl groups, as shown in Figure 2A. The hypertrophy of myocardial was evaluated by the protein expression of ANP and β‐MHC, which were indicator of hypertrophy, the protein level were all increased significantly in ISO group, and KN‐93 with a highest expression of ANP and β‐MHC, as shown in Figure 2C–E. The results indicated that KN‐93 promoted myocardial hypertrophy and interstitial fibrosis.

Results of Masson staining indicated that left ventricular collagen content in KN‐93 rats was much more increased compared with ISO and Ctrl, and CVF was increased obviously by KN‐93 compared with Ctrl and ISO, as shown in Figure 2B and 2F. The expression of collagen Ⅰ and Collagen Ⅲ were upregulated in ISO and were highest in KN‐93 group, as shown in Figure 2G,H. The results confirmed that KN‐93 promoted collagen deposition and aggravated myocardial interstitial fibrosis.

### 3.3 KN‐93 aggravated apoptosis of myocardial cells

**FIGURE 3:**
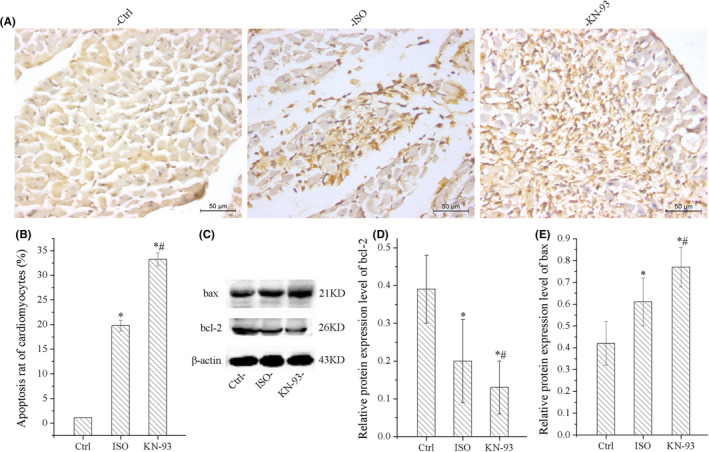
Results of TUNEL staining demonstrated that KN‐93 aggravated apoptosis of cardiomyocytes. (A) Representative image of TUNEL staining. (B) KN‐93 increased apoptosis rate of cardiomyocytes compared with ISO and Ctrl. (C) Representative bands of Western blotting of bax and bcl‐2. (D) KN‐93 down‐regulated bcl‐2 compared with ISO and Ctrl. (E) KN‐93 up‐regulated protein expression level of bax. All *p < 0.05 versus Ctrl, ^#^ *p *< 0.05 versus ISO. Error bars represent SD

The results of TUNEL staining indicated that KN‐93 significantly increased the number of TUNEL‐positive cells compared with ISO and Ctrl, as shown in Figure 3A, B. The protein expression of bcl‐2 was down‐regulated and bax was up‐regulated by KN‐93 compared with ISO and Ctrl, although ISO obvious induced apoptosis, increased expression of bax and decreased expression of bcl‐2 compared to Ctrl. The results were consistent with TUNEL, as shown in Figure 3B–E.

### 3.4 KN‐93 aggravated changes of myocardial ultrastructure in ISO rats

**FIGURE 4:**
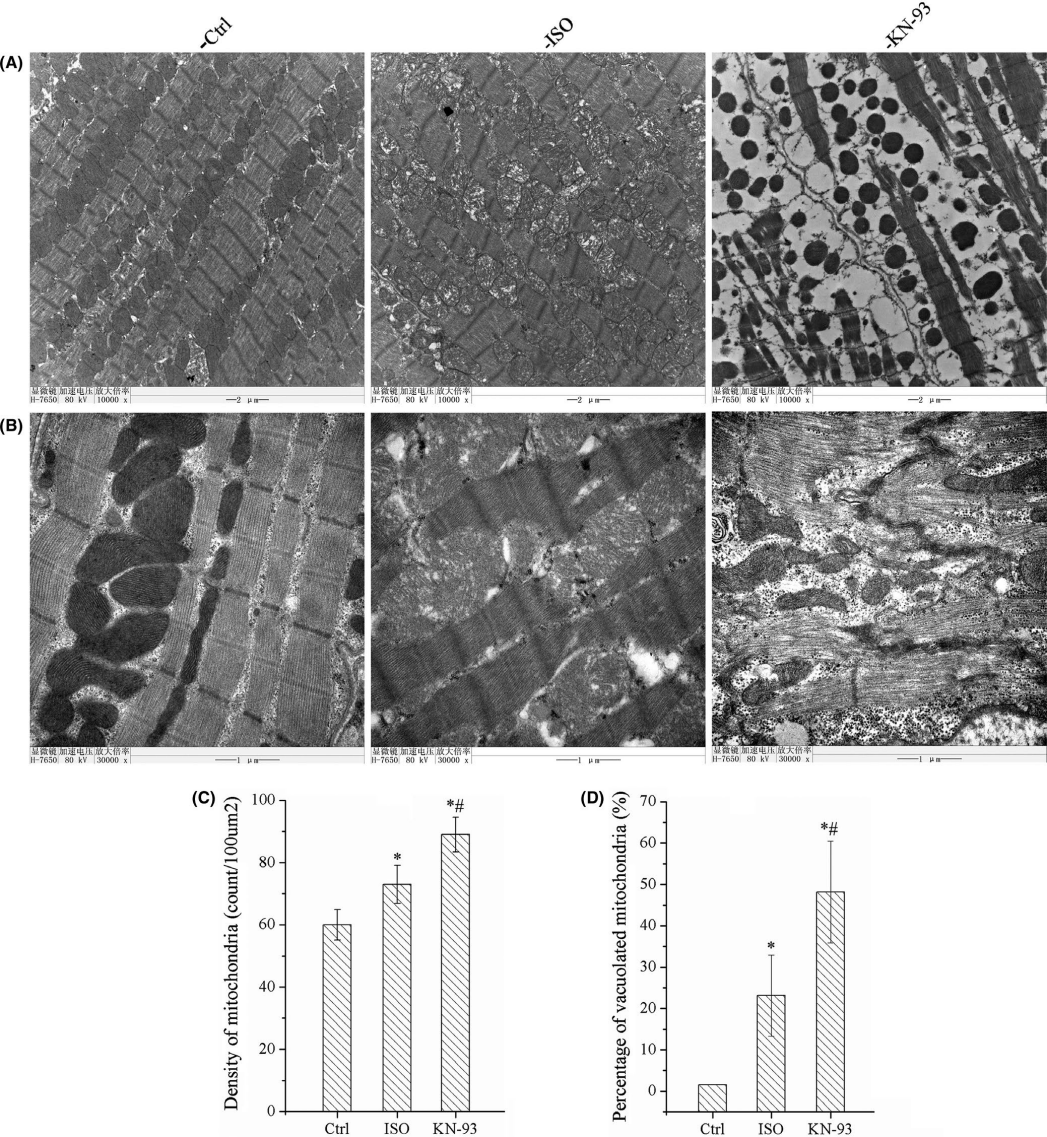
KN‐93 aggravated the ultrastructure of cardiomyocytes. (A) Representative ultrastructural images of TEM at low power (10000×). (B) Representative images of TEM at high power (30000×). All showed denaturation, oedema and destruction in many myofibrils, enhanced mitochondrial density, and a large number of mitochondria were damaged, collapsed or clumping and dissolved into vacuole. (C) KN‐93 increased density of mitochondria compared with ISO and Ctrl. (D) KN‐93 increased percentage of vacuolization in mitochondria compared with ISO and Ctrl. All *p < 0.05 versus Ctrl, ^#^ *p *< 0.05 versus ISO. Error bars represent SD

Myocardial ultrastructure was observed and analysed by transmission electron micrographs. The myocardial fibres were arranged regularly, the sarcomere structure was clear, mitochondria were neatly arranged, showed uniform morphology and intact structure in Ctrl group. In ISO group, myocardial cells were arranged in a disorderly manner, the plasma membrane of the myocardium was blurred, and the muscle filaments were loose, fragmentation and swelling, with absence of the Z line, increase of the mitochondria, mitochondria were varied in size and not neatly arranged, structure were unclear, swelling and crista fragmentation, or dissolved into vacuole. However, KN‐93 aggravated the changes of myocardial ultrastructure compared to ISO group, we observed severe denaturation, oedema and destruction in most of myofibrils, although enhanced mitochondrial density, but majority of mitochondria were damaged, they were collapse or clumping and dissolved into vacuole. The statistic results of density of mitochondria and percentage of vacuolization in mitochondria were represented as statistical histograms as shown in Figure 4.

### 3.5 KN‐93 reduced MVD, caused apoptosis of vascular endothelial cells and increased prevascular fibrosis ratio in myocardial tissue

**FIGURE 5:**
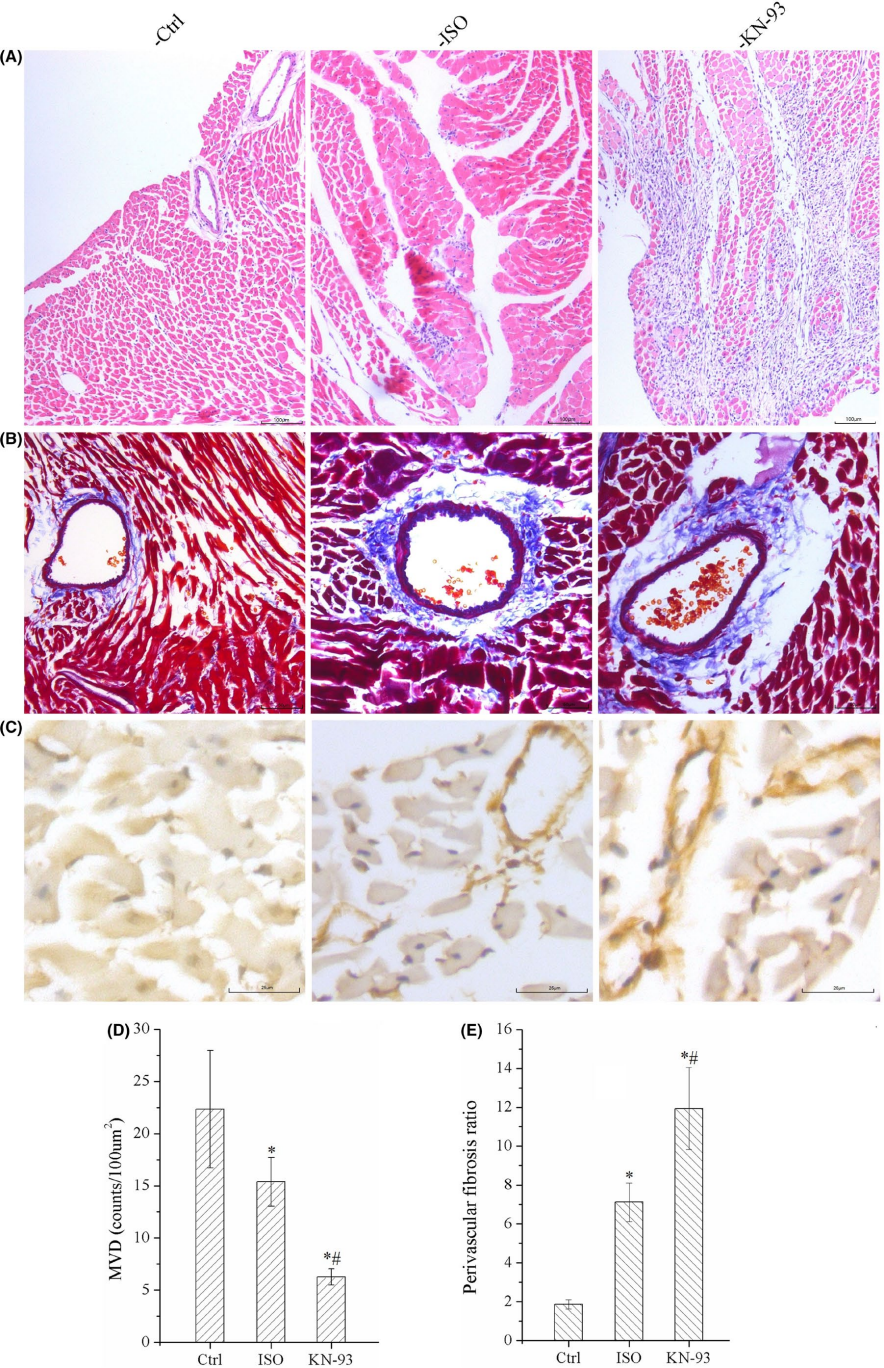
KN‐93 reduced MVD, promoted apoptosis of vascular endothelial cells, and increased prevascular fibrosis ratio in myocardial tissue. (A) Image of microvessel in HE staining. (B) Image of prevascular fibrosis in Masson's trichrome staining. (C) Image of apoptosis of vascular endothelial cells in TUNEL staining. (D) KN‐93 reduced MVD compared with ISO and Ctrl. (E) KN‐93 increased prevascular fibrosis ratio compared with ISO and Ctrl. All *p < 0.05 versus Ctrl, ^#^ *p *< 0.05 versus ISO. Error bars represent SD

The MVD and prevascular fibrosis ratio were measured, the statistic data indicated that MVD was much more reduced in myocardial tissue by KN‐93 compared with ISO and Ctrl, prevascular fibrosis also was aggravated by KN‐93, and we also observed apoptosis of vascular endothelial cells, as shown in Figure 5.

### 3.6 KN‐93 suppressed proliferation, migration and tubule formation of HUVECs

**FIGURE 6:**
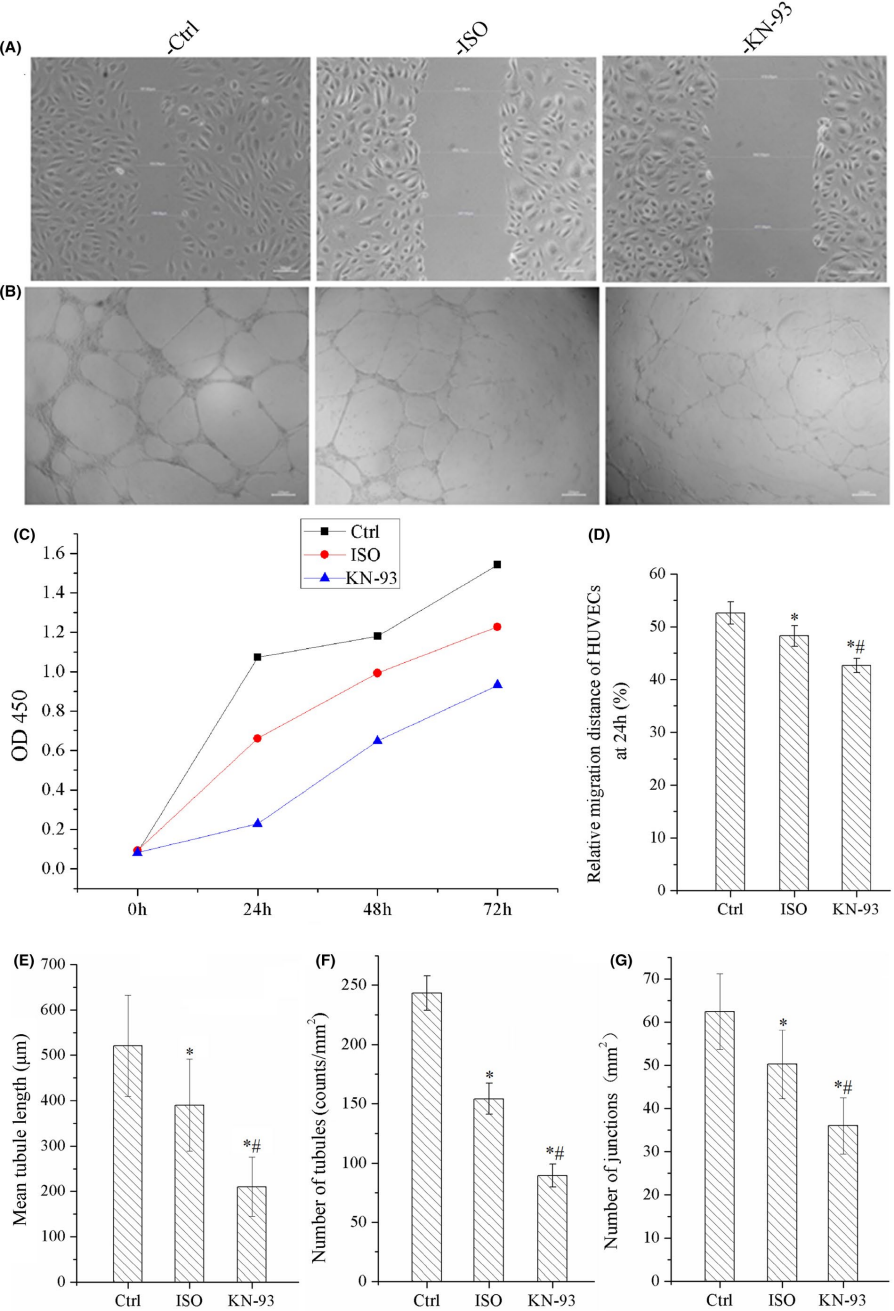
KN‐93 suppressed proliferation, migration and tube formation of HUVECs. (A) Image of migration of HUVECs. (B) Image of tube formation of HUVECs. (C) KN‐93 suppressed proliferation compared with ISO and Ctrl. (D) KN‐93 decreased relative migration distance compared with ISO and Ctrl. (E) KN‐93 decreased mean tubule length compared with ISO and Ctrl. (F) KN‐93 decreased number of tubules compared with ISO and Ctrl. (G) KN‐93 reduced number of junctions of HUVECs compared with ISO and Ctrl. All *p < 0.05 versus Ctrl, ^#^ *p *< 0.05 versus ISO. Error bars represent SD

We found that 1 uM KN‐93 had no effect on migration and proliferation of HUVECs; however, there was no tubule formation in cells treated with 50 uM KN‐93(as shown below) and 50 uM KN‐93 obviously induced cell death. The results of 10 uM KN‐93 were adopted. The relative migration distance of the cells was shortened by ISO, but was more shortened by KN‐93, as shown in Figure 6A, D. That is to say, KN‐93 suppressed the migration of HUVECs.

Figure 6C indicated that ISO reduced the cell survival; however, the cell survival was lowest in KN‐93 group. It indicated that KN‐93 severely inhibited cell survival of HUVECs.

The results of tubule formation assay showed that KN‐93 dramatically reduced the number of tubules, mean tubules length and number of junctions compared with Ctrl group and ISO group, as shown in Figure 6B, E–G. So, it significantly inhibited tubule formation of HUVECs.

### 3.7 KN‐93 promoted apoptosis of HUVECs

**FIGURE 7:**
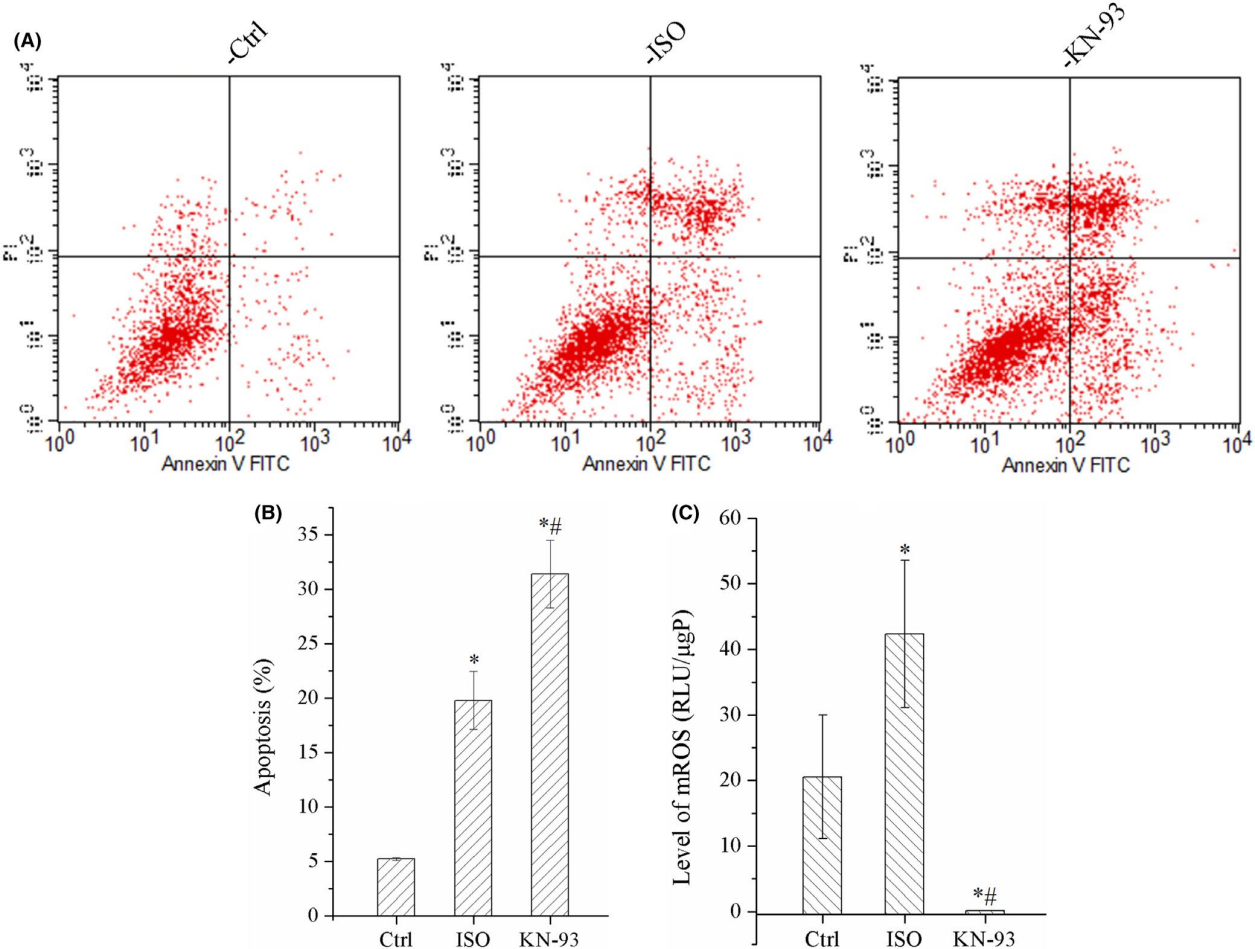
KN‐93 increased apoptosis and inhibited mtROS of HUVECs. (A) Image of flow cytometry. (B) KN‐93 increased apoptosis of HUVECs. (C) KN‐93 significantly reduced level of mtROS. All *p < 0.05 versus Ctrl, ^#^ *p *< 0.05 versus ISO. Error bars represent SD

The data of flow cytometry indicated that the number of apoptotic cells was the highest in KN‐93 group; it demonstrated that KN‐93 evidently promoted apoptosis of HUVECs as shown in Figure 7A, B.

### 3.8 KN‐93 severely limited the production of mtROS

The level of mtROS in ISO group was obviously increased; however, KN‐93 severely reduced the mtROS compared with Ctrl group and ISO group, as shown in Figure 7C.

### 3.9 KN‐93 down‐regulated the protein expression of NOX2, NOX4, p‐VEGFR2 and STAT3 in HUVECs

**FIGURE 8:**
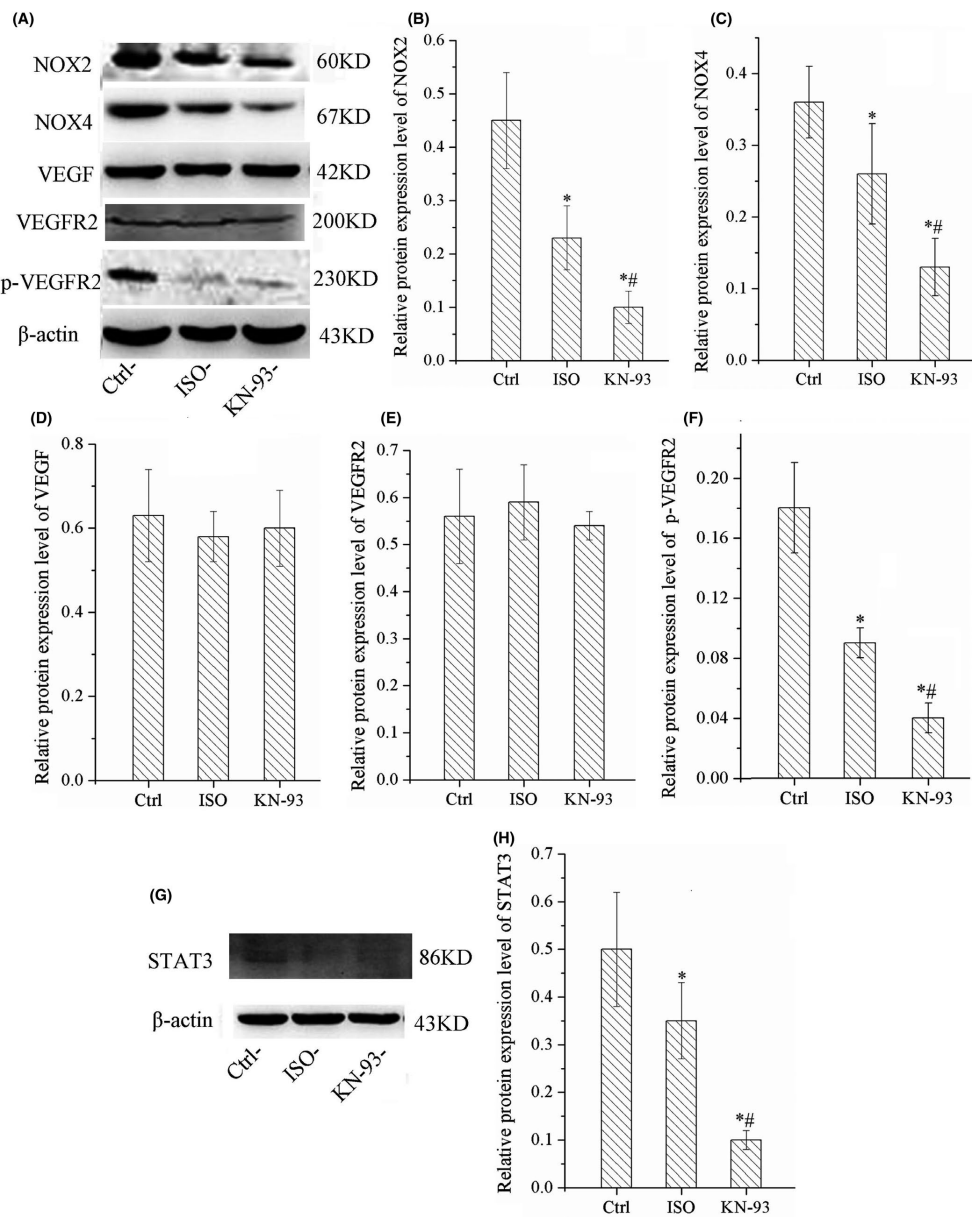
KN‐93 down‐regulated protein expression of NOX2, NOX4, p‐VEGFR2 and STAT3 in HUVECs. (A) Representative bands of western bloting of NOX2, NOX4, VEGF, VEGFR2, p‐VEGFR2. (B)(C). KN‐93 down‐regulated protein expression level of NOX2 and NOX4. (D)(E). KN‐93 had no effect on protein expression of VEGF and VEGFR2. (F) KN‐93 down‐regulated protein expression of p‐VEGFR2. (G) Representative bands of western bloting of STAT3. (F) KN‐93 down‐regulated protein expression of STAT3. All *p＜0.05 versus Ctrl, ^#^ *p*＜0.05 versus ISO. Error bars represent SD

WB results showed that ISO decreased the expression of NOX2, NOX4, p‐VEGFR2 and STAT3; however, these expression of proteins were more significantly down‐regulated by KN‐93, especially p‐VEGFR2 and STAT3, as shown in Figure 8. However, there was no change in expression of VEGF and VEGFR2. It suggested that p‐VEGFR2 was the pivotal molecule in angiogenesis.

## 4 DISCUSSION

Angiogenesis was a physiological and pathophysiological process that initiated vascular growth from pre‐existing blood vessels in response to a lack of oxygen. In recent years, therapeutic angiogenesis had been shown to revascularize ischemic heart tissue, reduced the progression of tissue infarction, and evaded the need for invasive surgical procedures or tissue/organ transplants,^34^ and it was considered as a potential treatment for ischemic heart disease (IHD) and was widely studied. Therefore, researchers had done a lot of exploration to reveal the correlation angiogenesis and cardiac remodelling and HF, including cardiac hypertrophy, fibrosis and heart function, they found that cardiac hypertrophy, remodelling and HF were inhibited by promoting myocardial angiogenesis,^17^, ^35^ and cardiac fibrosis also had a negative correlation with cardiac angiogenesis.^36^, ^37^ Similarly, some strategies which impaired angiogenesis thus promoted or aggravated ventricular remodelling and cardiac dysfunction.^20^, ^21^, ^38^ So, angiogenesis should be protective against cardiac remodelling and HF.

CaMKII was an important mediator for the ischaemia‐induced coronary angiogenesis.^13^ However, persistent cardiac CaMKII activation was considered to promote heart failure development,^3^ and some studies believed that CaMKII was a target for the treatment of heart failure.^39^ Whether inhibition of CaMKII could reverse or aggravate cardiac remodelling confused us. In this study, we demonstrated in vivo and in vitro that inhibition of CaMKII by KN‐93 impaired myocardial angiogenesis and aggravated cardiac remodelling and HF for the first time.

In tumour tissue, cell apoptosis was induced by inhibiting angiogenesis,^40^ and cardiac fibrosis had a negative correlation with cardiac angiogenesis^36^, ^37^; in this study, we demonstrated KN‐93 suppressed myocardial angiogenesis, thereby cardiomyocyte apoptosis and cardiac fibrosis were all aggravated by KN‐93, and the results were consistent with the previous studies.

We further revealed the underlying mechanism of action of KN‐93. VEGF induced angiogenesis by stimulating EC migration and proliferation primary through the VEGFR2, and VEGF/VEGFR2 was a classical pathway to regulate angiogenesis in many tissue.^41^ However, mtROS was an indispensable factor to active this pathway, VEGFR2 had to be phosphorylated by mtROS and activated, and the production of mtROS was promoted by NOX2 and NOX4, NOX2 sensed Nox4‐derived H_2_O_2_ to promote mtROS production and caused tyrosine phosphorylation of VEGFR2 (p‐VEGFR2) and angiogenesis, the production of mtROS and phosphorylation of VEGFR2 were suppressed when NOX2 was blocked.^23^ We found that KN‐93 significantly down‐regulated expression of NOX2 and NOX4, thereby markedly reduced the production of mtROS and p‐VEGFR2, but KN‐93 had no effect on expression of VEGF and VEGFR2. The results proved that KN‐93 inhibited HUVECs tubule formation via reducing production of mtROS. In addition, STAT3 was also a crucial factor to promote angiogenesis; we found that STAT3 was also down‐regulated by KN‐93. However, mtROS excessive accumulation could inhibit STAT3; this accounted for the suppression of angiogenesis in ISO group, which induced excessive emission of mtROS.^42^ So, we concluded that KN‐93 impaired myocardial angiogenesis via inhibiting NOX2/mtROS/p‐VEGFR2 and STAT3 pathways.

We also found by TUNEL staining that KN‐93 caused apoptosis of vascular endothelial cells, and this was confirmed in cell experiments, KN‐93 significantly induced apoptosis of HUVECs, probably because STAT3 was a protective factor of apoptosis,^43^, ^44^ but KN‐93 significantly down‐regulated the expression of STAT3, thus, apoptosis of vascular endothelial cells was promoted and angiogenesis was impeded. So, it created a vicious circle.

So, inhibition of CaMKII by KN‐93, which was a widely used inhibitor of CaMKII, impaired myocardial angiogenesis and aggravated cardiac remodelling and HF via inhibiting NOX2/mtROS/p‐VEGFR2 and STAT3 pathways. This study provided evidence for us to further understand the mechanisms of cardiac remodelling and HF.

In the end, our study has certain limitations, we only study KN‐93, there are other inhibitors of CaMKII, such as KN‐62/CK59 or some novel inhibitor, tatCN2, RA360 and so on. Perhaps, some of them can promote angiogenesis and be benefit to cardiac remodelling, and are worth further study.

## CONFLICT OF INTEREST

The authors declare that they have no competing interests.

## AUTHOR CONTRIBUTIONS

**Yajuan Ni:** Conceptualization (equal); Formal analysis (equal); Funding acquisition (equal); Methodology (equal); Project administration (equal). **Jie Deng:** Data curation (equal); Methodology (equal); Supervision (equal); Visualization (equal). **Hongyuan Bai:** Data curation (equal); Methodology (equal); Software (equal). **Chang Liu:** Investigation (equal); Project administration (equal); Validation (equal). **Xin Liu:** Software (equal); Validation (equal); Visualization (equal). **Xiaofang Wang:** Investigation (equal); Resources (equal); Visualization (equal).

## Data Availability

The data used to support the findings of this study are available from the corresponding author upon request.
